# Age-Based Dynamics of a Stable Circulating Cd8 T Cell Repertoire Component

**DOI:** 10.3389/fimmu.2019.01717

**Published:** 2019-08-06

**Authors:** Elena N. Naumova, Maryam B. Yassai, Wendy Demos, Erica Reed, Melissa Unruh, Dipica Haribhai, Calvin B. Williams, Yuri N. Naumov, Jack Gorski

**Affiliations:** ^1^Friedman School of Nutrition Science and Policy, Tufts University, Boston, MA, United States; ^2^Versiti Wisconsin, Blood Research Institute, Milwaukee, WI, United States; ^3^Department of Pediatrics, Medical College of Wisconsin, Milwaukee, WI, United States; ^4^University of Massachusetts Medical School, Worcester, MA, United States

**Keywords:** human CD8 T cells, computational immunology, repertoire maturation, circulation as depot, senescence

## Abstract

T-cell memory to pathogens can be envisioned as a receptor-based imprint of the pathogenic environment on the naïve repertoire of clonotypes. Recurrent exposures to a pathogen inform and reinforce memory, leading to a mature state. The complexity and temporal stability of this system in man is only beginning to be adequately described. We have been using a rank-frequency approach for quantitative analysis of CD8 T cell repertoires. Rank acts as a proxy for previous expansion, and rank-frequency, the number of clonotypes at a particular rank, as a proxy for abundance, with the relation of the two estimating the diversity of the system. Previous analyses of circulating antigen-experienced cytotoxic CD8 T-cell repertoires from adults have shown a complex two-component clonotype distribution. Here we show this is also the case for circulating CD8 T cells expressing the BV19 receptor chain from five adult subjects. When the repertoire characteristic of clonotype stability is added to the analysis, an inverse correlation between clonotype rank frequency and stability is observed. Clonotypes making up the second distributional component are stable; indicating that the circulation can be a depot of selected clonotypes. Temporal repertoire dynamics was further examined for influenza-specific T cells from children, middle-aged, and older adults. Taken together, these analyses describe a dynamic process of system development and aging, with increasing distributional complexity, leading to a stable circulating component, followed by loss of both complexity and stability.

## Introduction

Adaptive immune memory to pathogens arises by selecting particular lymphocyte clones from a pre-existing repertoire of naïve cells whose clonal antigen receptor has sufficient avidity to recognize the pathogen and initiate a response. Naïve lymphocytes show a high degree of species richness owing to the somatic rearrangement undergone by their antigen-specific receptor genes during their thymic development. Limited expansion of the thymocytes results in a relatively uniform naïve T cell frequency distribution, hence overall low diversity. Naïve cells are selected on pathogen in the periphery, and a portion of the expanded antigen-experienced cells are retained to guard against exposure to the same or similar pathogens. The original recruitment of adaptive immunity in a response to pathogen is based on antigen presentation by innate immune cells that are primary responders and which play a predominant role in clearing the first exposure.

Upon re-exposure by the same or similar pathogen, immature adaptive memory will still be augmented by an innate inflammatory response, which will constitute the signal for continuing maturation of the adaptive system. Mature functional adaptive memory can be defined as the point when the innate cells are primarily required the antigen presentation and not the inflammatory response. This maturation process may require multiple exposures or immunizations against some pathogens. An early elegant description of the maturation process for B cells came from the work of Berek and co-authors, who showed the appearance of new, mutated IgH genes encoding high affinity antibodies only after multiple immunizations to the same antigen (1, 2).

T cell responses do not undergo the mutational maturation as do B cell responses and the primary evidence for maturation is the expansion in the number of cells involved in the response. Antigen experienced T cells are characterized by differential expression of activation and homing molecules (3). Further studies have established concepts such as effector vs. central memory (4, 5) and importantly the movement of memory T cells into depots and tissues (6–8). Analyses of immune and tissue depots have been recently described in man (9, 10). Memory maturation in humans has the added dimension of thymic involution (11) at puberty, which limits the number of new clonotypes that can enter the adult memory pool (12).

Our studies of the CD8 memory T cell repertoire take advantage of cellular expansion. The precursor frequency of the cells in the circulation is increased after expansion and the cells continue to show a strong replicative/survival response in culture when stimulated by a pathogen-derived peptide epitope. The latter can be considered as an additional *in vitro* exposure. Our focus has been on the CD8 T cell response to the conserved, matrix-derived, influenza epitope, M1_58−66_. In individuals positive for human leukocyte antigen A2 (HLA-A2), this peptide drives a complex recall response. The distribution of the cells can be described as composed of two components when analyzed by rank frequency analysis (13). The first component is power law-like and the second component is composed of higher-ranking clones with typically only one exemplar per rank. The repertoire is characterized by use of the TRBV19 gene (hereafter referred to as BV19) which encodes Arg and Ser as part of the non-germ line component of the third complementarity determining region (CDR3) of the receptor (14–16). The CDR3 length is 11 amino acids and the RS appears at position 5. We have shown that the same complex clonotype distribution holds whether the cultures are further subdivided by their cytotoxicity, cytokine secretion, or binding of major histocompatibility complex (MHC)-bound antigen multimers (17). We have also shown that the distribution of the two components changes between middle-aged and older subjects (18). Recently, we have shown that the entire circulating CD8 BV19 repertoire, which subsumes the flu-specific repertoire, shows the same two component rank-frequency distribution as observed in the recall repertoire (19).

The first, power law-like, component of the distribution reflects the action of a repeated birth-death selection process (20, 21). It can also be viewed as affinity-based selection for replication of a set of cells that are initially normally distributed with respect to affinity for a ligand (13). The second component of the MI_58−66_-specific and BV19-specific repertoires has posed a puzzle as to its significance. It could represent a secondary selective expansion process. However, a simpler explanation is that the second component reflects a differential abundance of well-selected clonotypes in the circulation. We therefore expect that such clonotypes, in addition to being sampled at higher than expected frequencies, will be stable over time. We also expect that the second component will be a function of age as it is unlikely a stable repertoire component can precede the establishment of the initial complex repertoire.

Here we use a measure of clonotype stability of circulating BV19 clonotypes from five adult subjects to show that the second distributional component is indeed stable. We then go in to show the same relation can be observed for recall repertoires. Furthermore, the circulating stable component is not observed in children, and is present in a degraded form in older adults. The results are discussed in terms of repertoire development and senescence. The significance of a circulating pool of CD8 T cells is also discussed.

## Materials and Methods

### Study Cohorts

Peripheral blood mononuclear cells (PBMC) were collected from five healthy child subjects (C1, C2, C3, C4, and C5), six healthy middle-aged adult subjects (mA1, mA2, mA3, mA4, mA5, and mA6) and six older adult subjects (oA1, oA2, oA3, oA4, oA5, and oA6). All subjects were typed as HLA-A2.1-positive. Ages at time of enrollment, number of blood samples and average time span between samples for the *ex vivo* sequencing and *in vitro* recall studies are provided in Table 1. The timing of sample collection relative to the date of first sampling is provided in Supplemental Table 1 and illustrates the spacing between individual measurements and general overlap across study subjects. This timing data shows that our estimates of stability are derived under similar conditions for each person. Because we are interested in steady state conditions, samples were used from time periods during which the subjects did not report flu-like illness since the previous sampling. A subset of adult subjects performed bi-weekly self-administered swabs during the local the flu season. The samples used here were not taken from samples collected after a swab positive for influenza.

**Table 1:** Age and sample collection data of the study cohorts.

		**Subject ID**	**Age at first blood sample (in years)**	**Number of blood samples**	**Average time span between samples (in months)**
*Ex vivo* HTS	Adults	oA1	68	6	3.77
		mA1	39	7	2.94
		mA2	40	6	3.31
		mA3	40	5	2.55
		mA4	44	6	3.37
		Average^§^	46.20 ± 12.34	6.00 ± 0.71	3.19 ± 0.46
Recall	Children	C1	7	8	3.79
		C2	9	5	7.98
		C3	10	6	3.77
		C4	12	8	3.79
		C5	14	7	3.79
		Average	10.40 ± 2.70	6.80 ± 1.30	4.62 ± 1.88
	Middle-aged adults	mA1	39	10	2.65
		mA2	40	8	3.44
		mA5	40	8	2.49
		mA6	48	10	4.43
		Average	41.75 ± 4.19	9.00 ± 1.15	3.25 ± 0.89
	Older adults	oA1	68	8	1.94
		oA2	78	8	5.69
		oA3	69	9	3.56
		oA4	78	13	3.81
		oA5	80	5	5.36
		oA6	78	8	4.56
		Average	75.13 ± 5.23	8.50 ± 2.59	4.15 ± 1.37

§ *Indicates mean ± standard deviation*.

The healthy child subjects were enrolled under protocol Children's Hospital of Wisconsin IRBnet: 116305 “Generation and decay of memory T cells in children with Juvenile Rheumatoid Arthritis and healthy siblings following administration of trivalent inactivated influenza vaccine,” from the Children Hospital of Wisconsin. The subjects analyzed here were the controls in this study. The adult subjects were enrolled under protocols authorized by the Institutional Review Board of BloodCenter of Wisconsin: BC 05-11, “Generation and Decay of Memory T Cells in Older Populations,” and BC 04-22, “Robust T Cell Immunity to Influenza in Human Populations.” These protocols have been transferred to the IRB of the Medical College of Wisconsin (MCW). Written informed consent was obtained from participants, or their parents/legal guardians in the case of children.

### M1_58−66_ Recall Culture and Clonotyping

PBMC were isolated using Ficoll-Paque plus (Amersham Biosciences) and stored frozen under liquid N_2_ until used. The M1_58−66_ peptide (GILGFVFTL) from the M1 protein of influenza A virus was synthesized by The Blood Research Institute Peptide Core. The procedure for the culturing PBMC, CD8 cell selection, nucleic acid preparation, amplification, cloning, and sequencing has been described previously (17). The recall analyses were performed as part of our general human immunology studies. PBMC were stimulated at 1 × 10^6^ cell/ml in 2-ml cultures with M1_58−66_ peptide added to 1 μM final concentration in complete RPMI media supplemented 10 U/ml of recombinant IL2 and 10% human pooled AB sera in round bottom tubes or wells for 7 days. On day 3, an IL2 supplement (10 U/ml) was provided. On day 7 non-adherent cells were collects after agitation, counted and re-plated with an equivalent number of fresh irradiated autologous PBMC at 10^6^ cells/ml. The feeders had been prepulsed with peptide (1 μM final concentration). IL2 was added to 10 U/ml. Another 7-day culture was performed with IL2 addition at day 3. However, the analysis for subject mA6, and for most of the child samples was performed with our dendritic cells (DC) protocol in which adherent cells are prepared by overnight culture. Half of these adherent cells (monocyte derived APC) are used for the first week stimulation of the non-adherent PBMC (i.e., lymphocytes), and the other half maintained in IL4 and GM-CSF for use in the second week. All cultures in adults were in triplicate, and predominantly in duplicate for the child cohort owing to smaller blood sample volumes.

After two 7-day cycles of recall culture, CD8 cells are isolated by magnetic beading using Dynal CD8 positive isolation kit (Invitrogen Inc., Carlsbad, CA) according to manufacturer's instruction. mRNA samples were isolated from the CD8 cells using Dynal Oligo (dT) beads according to manufacturer's instructions (Invitrogen). cDNAs was prepared using a poly-T primer and MMLV reverse transcriptase (Invitrogen). All cDNAs were titrated using a pair of C-region primers, one labeled with fluorescein, in three PCR reactions for 20 cycles, each reaction using a doubling of the cDNA concentration. The cDNA for BV19 analysis was used at the concentration corresponding to the midpoint in the linear plot of cDNA concentration to amplicon fluorescence intensity using cDNA concentrations where the amplicon fluorescent intensity increased in direct relation to the cDNA concentration. The PCR used our standard BV19 and BC primers (22). The BV19 primer concentration was 20 times the concentration of the C-region primers used in the titration to ensure the same efficiency. As long as the experiments are performed under these conditions, they should provide representative data about the sample. Since all samples obtained from humans are far from exhaustive, representative data is all that can be expected.

We chose CD8 selection after having examined the outcomes of separating the cells based on CD107 expression as a marker for degranulation/cytotoxicity function and M1_58−66_:HLA-A2 multimers as a TCR affinity marker. We observed that each of these showed a complex repertoire, but they were not completely overlapping. Hence CD8 represented the broadest selection and was the simplest to use as well (17), which is important when large number of samples are involved.

The PCR product was cloned into *E. coli* using pCR4-TOPO Cloning Kit (Invitrogen, Carlsbad, CA). Bacterial colonies (~400) were grown overnight and sent to Agencourt Bioscience (Beverly, MA) for sequencing. Sequences were received in *fasta* format and analyzed using “CDR3Reader” software, which identified V and J regions, assigns clonotype names according to our convention (23), and counts occurrences of each clonotype. The identity of a distinct instance of a β-chain is based on the rearrangement site with respect to each of the two rearrangements that generated the chain, D to J and V to DJ. The region between the sites is referred to as the NDN region which represents the junctional diversity present in all the β-chain genes that underwent the same D to J and V to DJ choice. The NDN region is embedded in the CDR3 (24), which is composed of all the amino acids between the conserved cysteine at the c-terminus of the V gene and the conserved phenylalanine-glycine in the J region. The naming convention provides the information as to which V and J regions were used, the sequence and encoding of the NDN as well as the length of the CDR3.

Data analyzed represents pooling of the duplicate and triplicate cultures. Although the colony counting procedure involves ligation and bacterial transformation steps, the results are reproducible as tested in experiments in which large cultures were divided in three and each portion subject to CD8 selection, bacterial cloning and sequencing. There was an excellent clonotype overlap between the three separate assays of the same culture [Supplemental Figure 1 in (17)].

It should be pointed out that our definition of clonotype is only based on the TCR β-chain. Most T cells only express one β-chain, referred to as allelic exclusion, so this is a close one to one mapping. However, after thymic β-selection, the DN thymocytes expand prior to α-chain gene rearrangement (25, 26). Thus, cells with the same β-chain may have different α-chain partners, with cells with each distinct β-α pair representing a separate clonotypic lineage. Thus, our description of diversity is an underestimate, as our analysis would group all of these as one lineage.

### High Throughput Sequencing (HTS) of BV19 TCR

T cell sequence analysis is described in more detail elsewhere (19, 27), including error estimation, and steps taken in cleaning the nucleotide sequence data, defining motifs and motif distributions. In brief, PBMC from five to seven different time points per subject were used (Table 1, *ex vivo* HTS panel). PBMC were thawed, CD8 cells collected by magnetic bead separation and mRNA and cDNA prepared as described above. PCR amplification was done using our standard BV19 and BC primers modified to include the Roche 454 adapter sequences and sample ID tag sequences. Owing to the higher concentrations needed for 454 sequencing in lieu of scaling up, multiple amplifications were performed, each equivalent to the reactions used for the cultures. The concentration of purified PCR products was measured using NanoDrop-1000 spectrophotometer. From 6 to 12 purified PCR products were pooled to obtain a total of 2,500 ng. The samples were further amplified and prepared for high throughput sequencing at the Human and Molecular Genomic Center (HMGC) Sequencing Facility (www.hmgc.mcw.edu) of Medical College of Wisconsin. The sequencing was performed on the Roche GS-FLX Genome Sequencer using Titanium chemistry. Samples were coded by identifier sequences embedded in the primers. After decoding, sequences derived from each sample were downloaded in *fasta* format and analyzed using “CDR3Reader.”

The HTS data differs from the recall data in the presence of two power law-like components in the rank-frequency analyses. The method used did not include a unique molecular identifier as part of the cDNA or second strand synthesis (28). With the additional amplification associated with Roche 454 sequencing it is very likely that the shift to higher ranks of the second component is associated with the concentration of cDNA (sample) being analyzed and the number of amplification cycles. Decreasing the concentration of cDNA under identical experimental conditions enhances the shift (unpublished), thus our analyses were restricted to using sufficiently high concentrations of cDNA that minimized this effect. This implies starting with a sample size sufficient to clearly observe the lower ranks.

### Data Analysis

The repertoire data from any sample can be tabulated as the clonotype name and the number of observations of that clonotype. Data from such a table can be used to define some key repertoire measures: number of clonotypes, *N*, number of observations, *M*, number of clonotypes observed once (i.e., singletons), *N*_*S*_, the highest ranking clonotype, *Rmax*. Rank frequency analysis involves counting the number of clonotypes observed once, twice, thrice, to *Rmax*. These measures in turn can be used to generate a number of repertoire characteristics. We use: (1) *N* as a general proxy for richness, (2) $\frac{M}{N}$, observations per clonotype as a proxy for abundance, (3) $\frac{Ns}{N}$, the fraction of clonotypes observed once (i.e., rank = 1) to describe the singleton tail of the distribution, and (4) $\frac{Rmax}{M}$, the proportion of observations due to the highest ranking (i.e., most frequently observed) clonotype. These four characteristics offer a general overview of the distribution as they provide an average richness and abundance and a description of the two extremes.

We used a similar approach to clonotype temporal stability within the repertoire. The stability of the clonotype is defined as the number of times it was observed across repeated measurements. Clonotypes observed at all times analyzed are considered stable and clonotypes observed only at one time unstable. Because the measurement of temporal stability is based on multiple time points, more time points should provide a better estimation of stability. To compensate for small differences in the number of time points, we introduce a relative stability characteristic of the repertoire in which observation at one time is equal to a stability of 0, observation at all times is equal to a relative stability of 1. Thus, the relative stability of a clonotype is calculated as: $\frac{number\;of\;times\;observed\;-\;1}{maximum\;possible\;number\;of\;times\;observed\;-\;1}$. The average relative stability of all the clonotypes at a given rank is the average of all the individual relative stabilities of clonotypes at that rank. Thus, clonotypes with rank lower than six in the pooled repertoire that consists of six time points cannot be stable. To assess the relationship between clonotype distributional frequency and the estimates of temporal stability we used correlation analysis and provided correlation coefficients, *R* and coefficients of determination, *R*^2^.

While our data sets are of similar size, the number of observations (*M*), and clonotypes (*N*) can vary, we used a number of normalization procedures. Normalized rank can be used for plotting the relation to normalized rank frequency or to average relative stability. We normalize each *ln*-rank by dividing the log-transformed values of *Rmax*; the latter representing the largest rank possible. The extreme values in this case are 0 for $\frac{\ln\;1}{\ln\;Rmax}$ and 1 for $\frac{\ln\;Rmax}{\ln\;Rmax}$. For rank frequency, we normalize the *ln*-rank frequency by dividing by the highest frequency component which is when the rank = 1 (singleton clonotypes = *Ns*). This spreads the data from one to zero, with one reflecting the contribution of the highly abundant singleton clonotypes, resulting from $\frac{\ln\;Ns}{\ln\;Ns}$ = 1, and the frequency of the highest ranking clonotype (usually one) equal to zero; $\frac{\ln1}{\ln\;Ns}$ = $\frac{0}{\ln\;Ns}$ = *0*. This normalization procedure works best for comparison of power law-like distributions. The normalized rank and rank frequency relationships were formally tested using an anchored power-law regression model, in which we regressed normalized *ln*-rank frequency *y* against normalized *ln*-rank *x* as follows: *y* = 1 – (1 – (1 – *x*)^*u*^)^*v*^, where *u* and *v* are the power-law parameters that govern the relationship curvature.

The data collected here represent clonotype numbers and frequencies that were a function of the cDNA input used to generate the amplicons used for the subsequent Roche GS-FLX Genome Sequencer analysis. Increasing or decreasing the concentration of cDNA increase or decreases the number of clonotypes identified and the frequency of the low ranking clonotypes as well as the maximum rank. Data were analyzed using Microsoft Excel and RStudio. Our definition of “clonotype” as used here has been qualified above.

The clonotype datasets generated and analyzed here are available as Supplemental Data 1 as is the approach for deriving the CDR3 nucleotide sequence from the clonotype names (Supplemental Figure 1).

## Results

### Role of Clonotype Rank as a Proxy for Selection

A repertoire is composed of clonotypes, which are defined by the clonal rearrangement of the receptor genes. As the clonotype is peripherally selected it expands and thus has an increased frequency within the repertoire. The frequency is measured by the number of observations after a controlled amplification of the receptor genes or transcripts. As long as the frequency is attributed to the entity “clonotype,” the analysis of the repertoire is limited to counting the clonotypes and/or some characteristic thereof. A higher level of analysis is obtained if the repertoire is described by a frequency of frequencies. In this approach, the absolute or relative measurement of the clonotype defines the rank of the clonotype. Thus, the repertoire (a pathogen-specific ecosystem) can be viewed as a collection of clonotypes (species) whose previous successful selection defines their rank (abundance). We will be sampling this system indirectly, from the circulation, and our samples will represent a small portion of the overall repertoire. Therefore, our quantitation will be relative but should reflect proper relationships as long as we do not skew the counting process by the methodology used to amplify the signal. The methods section describes the precautions we take to be in the proper relation of starting cDNA and amplification cycles.

As we have previously described (13, 17, 18, 27), the rank-based description of the repertoire shows that the highest frequency of responding clonotypes is that of clonotypes representing the lowest rank, i.e., those measured once. A log-log transformation of the rank vs. rank-frequency data shows a two-component plot with one component decreasing in a linear manner, and the second consisting of a number of mostly single clonotypes at very high ranks. The first component is indicative of a power law-like distribution fitting the equation *y* = *ax*^*b*^. The exponent, *b*, describes the distribution of the rank frequency of the clonotypes in the repertoire as it descends from the lowest to highest ranks. In the log transformation of the above equation, log *y* = b^*^log x + *a*, parameter *b* represents the slope of the data which is approximately linear. Parameter *a* represents the proportion of clonotypes that constitute the lowest rank, and is the *y* intercept of the line.

**Figure 1:**
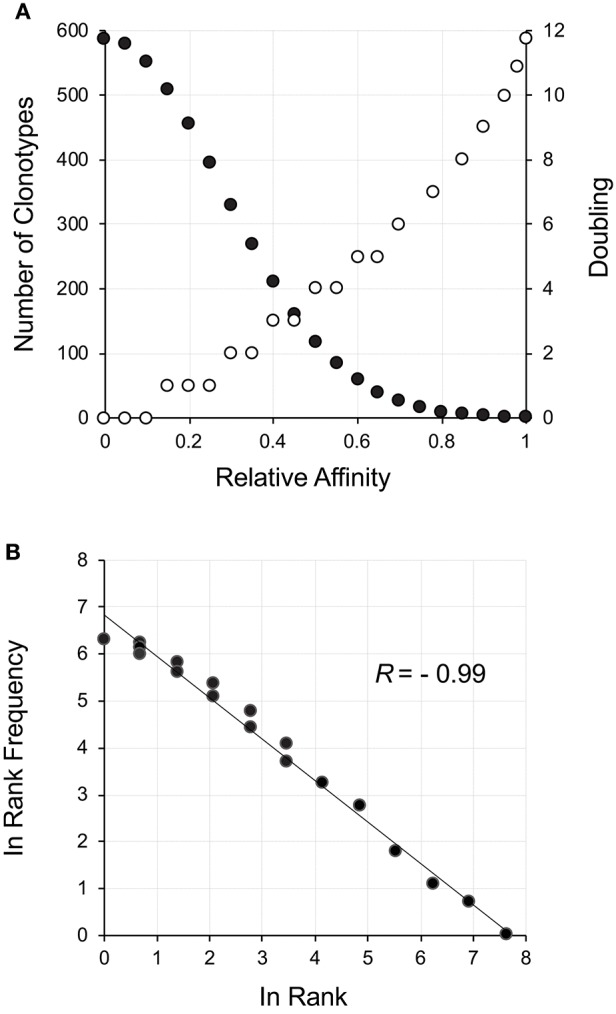
Power law-like distribution as a result of a reward function applied to a starting clonotype population normally distributed with respect to affinity. **(A)** The neutral to positive affinity portion of a normally distributed (σ = 0.14) population of clonotypes is shown as filled circles. The corresponding reward function resulting in the number of cell divisions is shown as empty circles. Since the division process is discrete, a range of affinities can fall within a particular doubling threshold. The reward function was set for a maximum of 12 divisions in equally distributed steps across the affinity spectrum. **(B)** The reward was applied to the number of cells at each affinity increment and the rank frequency of the resulting distribution calculated and plotted.

The thymus produces an initial repertoire that is relatively uniform, with skewing due to increased probability of certain aspects of the rearrangement mechanism (29) along with the limited expansion after β-selection (26, 30). Our initial description of how a power law-like distribution may arise from an initially uniform distribution was very focused on the TCR but actually represents a general phenomenon. With a random uniform distribution of receptors, a measure of affinity for a particular ligand will be normally distributed (31). A left-censored normally distributed distribution represents positive affinity with the maximum number of receptors being neutral and a small number representing the high affinity. An example for ~4,000 clonotypes representing a left-censored distribution is shown in Figure 1A (filled circles). All that needs to be done is to postulate that affinity will correlate with response, which includes cell division (32, 33). This can be thought of as a reward function for the lymphocyte network. A reward function resulting in 12 divisions (3–4 days) for the highest affinity (2^12^ ~ 8,000 cells) and no divisions, but survival, for neutral affinity, is shown (Figure 1A, open circles). The resultant repertoire distribution (Figure 1B) shows a power law-like distribution. Hence, an initial uniform distribution of clonotypes that display a normal distribution with respect to affinity to ligand can give rise to distribution with power law-like characteristics on the basis of selective cell division, with the clonotype rank describing the selection.

An important characteristic of a power law-like distribution is that it is scale free. From a sampling perspective, this means that doubling the amount of cells, will generate the same distribution pattern with some of the cells that were observed once now being observed twice, some that were observed twice now three times, etc. If we only use half the cells, we lose some singletons, some doubletons become singletons, etc. However, as long as the PCR cycle number is decreased, the distribution remains the same and is still representative. Without compensating the cycle number, the data becomes skewed owing to over-amplification.

However, the immune response is also characterized by a reduction of the expanded population after pathogen clearance, which we have modeled as a birth-death process (20, 21). The birth-death model more closely approximates our actual observations. Of course, even the birth-death model does not incorporate other factors like signaling thresholds, nor does it address possible probabilistic approaches to cell division which would require counting numbers of APC-T cell interaction, or numbers of exposures. However, it is clearly a guiding principle for arriving at a power law-like distribution from a uniform normal distribution and shows the usefulness of approaching repertoires using clonotype rank as a descriptor.

### Rank-Frequency Distribution of Adult BV19 Utilizing CD8 T Cells

Our initial analysis of complex repertoires utilized the recall response to influenza M1_58−66._ Circulating CD8 T cells expressing the BV19 TCR represent the next higher level of repertoire structure that encompasses this response. Analyzing the BV19 repertoire would represent a generalization of our findings. Indeed, an initial HTS analysis of BV19 CD8 TCR from an adult subject showed a similar complex clonotypic distribution to our previous recall data (19). Here we have added the HTS analysis of pooled circulating BV19 CD8 T cell repertoires from four middle-aged adult subjects, mA1 to mA4. The subject age and average sampling data are given in Table 1 (*ex vivo* HTS panel). Sample collection relative to the timing of the first sampling is provided in Supplemental Table 1. In all cases the pooling was of samples collected on average every 2 months, and the period of elapsed time between first and last samples is approximately a year and a half. Standard repertoire measures and characteristics for the pooled repertoires, described in the Method section, are provided in the top panel of Supplemental Table 2. The data from the five subjects differed slightly in depth of analysis at the level of number of observations, *M*. We also provide a summary of the repertoire measures and characteristics for the individual samples in the form of average values and deviations in the bottom panel of Supplemental Table 2. As might be expected, there was some variability in the measures obtained at the different time points for all subjects but overall the values are comparable.

**Figure 2:**
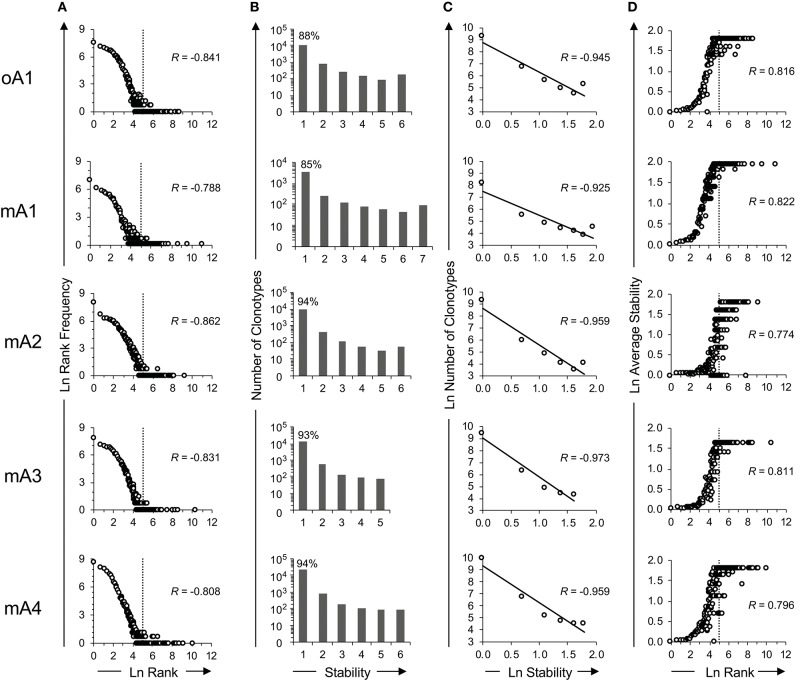
Time series analysis of the *ex vivo* BV19 repertoires of five adult subjects using high throughput sequencing. **(A)** Natural log-transformed clonotype ranks vs. rank frequency. The inflection point in the graph is identified by vertical dotted lines at *ln*-rank 5. **(B)** Repertoire stability data. The absolute number of clonotypes is shown for each stability increment. The clonotype count (y-axis) is on a log_10_ scale. The percentage of clonotypes observed at one time is shown above the bar graph. **(C)** The natural log-transformation of the data in panel B. **(D)** The log-transformed average stability is plotted as a function of *ln*-rank. The vertical lines show the two rank components defined by their inflection points of the distributional curve. The *R* values, where shown, describe the coefficient of correlation.

The rank frequency analysis for the pooled repertoire data for all five subjects is shown in Figure 2A. The rank frequency plots are very similar, and each has a power law like component and a second high-ranking component which are demarcated at rank *ln* 5 by a vertical line. Thus, *ln* 5 corresponds to the critical point dividing the power law-like component(s) from the high-ranking component. As noted previously (19), the power law-like component appears to have two parts which are divided at ~ *ln* 2. It should be pointed out that the data were generated without using unique molecular identifiers (28) and it is very likely that the two parts of the power law-like component observed in the rank-frequency analysis is a function of sample concentration to amplification cycle ratios. All five BV19 repertoires show a similar clonotype distributional frequency profile with the average correlation coefficient of −0.83 (*R* = −0.83 ± 0.03 and *R*^2^ = 0.68 ± 0.05). Individual *R* and *R*^2^ values are shown in Table 2, in the section labeled “Rank vs. Rank Frequency Figure 2A.”

**Table 2:** Coefficients of correlation (*R*) and determination (*R*^2^) between: (1) rank and rank frequency, (2) stability and number of clonotypes, and (3) rank and average stability for each individual within the study cohorts in reference to Figure 2.

	**Rank vs. rank frequency**		**Stability vs. number of clonotypes**		**Rank vs. average stability**	
	***R***	***R^**2**^***	***R***	***R^**2**^***	***R***	***R^**2**^***
**Subject ID**	Figure 2A		Figure 2C		Figure 2D	
oA1	−0.841	0.708	−0.945	0.893	0.816	0.665
mA1	−0.788	0.621	−0.925	0.855	0.822	0.676
mA2	−0.862	0.744	−0.959	0.921	0.774	0.599
mA3	−0.831	0.691	−0.973	0.946	0.811	0.658
mA4	−0.808	0.653	−0.959	0.92	0.796	0.633
Average^§^	−0.83 ± 0.03	0.68 ±−0.05	−0.95 ± 0.02	0.91 ± 0.03	0.80 ± 0.02	0.65 ± 0.03

§ *Indicates mean ± standard deviation*.

### Clonotype Stability

Clonotypes in a pooled repertoire have a measure describing the number of times they are present among the different sample times, which can define the stability of the clonotypes in the repertoire. This, measure is defined by the number of time points (increments) at which the clonotype was observed. With a pooled repertoire representing a number of distinct sampling times, a clonotype that is observed once is considered unstable and one observed at all times is considered stable. The temporal stability of the pooled repertoire is defined by the number or relative frequency of the clonotypes at each stability increment. The repertoire temporal stability data is shown for all five subjects in Figure 2B. The number of possible observation times (stability) is on the x-axis. The number of clonotypes at each stability increment is plotted on the y-axis using a logarithmic scale and the percentage of clonotypes observed only once is shown above the first bar. Most clonotypes are only observed once, indicating their temporal instability.

The repertoire stability is characterized by a decreasing frequency of clonotypes at higher stability increments. This was examined in more detail by plotting the natural logarithms of stability and clonotype frequency at each stability increment (Figure 2C) which showed that this relation is also power law-like. The value of the negative correlation between *ln* stability and *ln* number of clonotypes (*R* = −0.95 ± 0.02 and *R*^2^ = 0.91 ± 0.03) is very similar for all the subjects, irrespective of the variation in the numbers of times sampled or number of observations and clonotypes between the individuals. This similarity implies that we are defining a fundamental characteristic of the repertoire. The *R* and *R*^2^ values associated with Figure 2C and the means and standard deviation for these data sets are given in Table 2: Stability vs. Number of Clonotypes.

It was of interest to examine the relation of the stability measure with relation to clonotype rank. This is done by calculating the average stability for all the clonotypes observed at a particular rank. Figure 2D shows that stability increases as the rank increases. The *R* values are shown for each subject and the average of *R* = 0.80 ± 0.02. The *R*^2^ values are provided in Table 2 section “Figure 2D” and the average of *R*^2^ = 0.65 ± 0.03. For all five subjects, there is a rank after which the clonotypes are all stable although the extent of this fraction can vary from subject to subject.

### Comparison of Clonotype Rank Frequency and Stability as a Function of Rank

**Figure 3:**
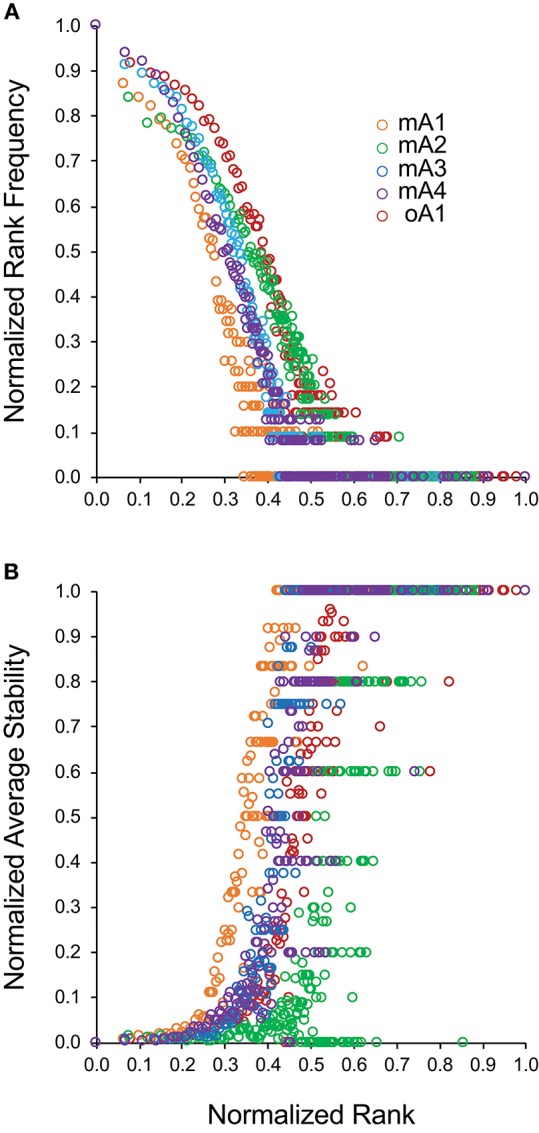
Relationship between normalized clonotype rank and either **(A)** normalized rank frequency or **(B)** normalized average stability for five adult subjects. Subject data is identified by different marker colors as shown in the insert of **(A)**.

Examining Figures 2A,D indicates that there may be a direct relation between rank-frequency and rank-stability. This relation was analyzed by generating a measure of normalized rank and plotting either normalized rank frequency (Figure 3A) or normalized average stability (Figure 3B) as a function of normalized rank. The normalized rank frequency plots for each subject differed slightly with respect to slope and inflection point between the second and third component. The stability plots showed the same relative differences resulting in a striking symmetry between the two data sets. Stability is a function of increasing rank which is inversely associated with frequency of clonotypes at that rank. There is some noise, defined as a spread of a particular stability level over a number of ranks, in the stability data. The significance of the noise, which is most apparent in the data from subject mA2, is still not clear. Overall, the data show that clonotype stability together with clonotype distribution are integral properties of overall repertoire complexity.

The stability measures and characteristics of the five pooled repertoires are provided in Supplemental Table 2 and Figure 2B. While the fraction of stable clonotypes varies between 0.004 and 0.021 (average ~0.01) this small fraction of stable clonotypes can represent an average of ~0.38 of the observations (proportion of stable clonotypes, $\frac{Mst}{M}$). Thus, a large part of the circulating BV19 CD8 T cells are composed of a small number of very stable clonotypes.

The results of these *ex vivo* analyses of adult CD8 repertoires show that there is a small percentage of clonotypes representing a large percentage of T cells that are represented in the circulation at all times, which is compatible with a stable circulating depot of cells. This also would explain the different distribution of the second rank-frequency component. For this component, rank is not just a function of previous expansion but also of accessibility.

### Stability of the M1_58−66_ Recall Repertoire in Subjects Representing Three Age Cohorts

The BV19 data reflects a comprehensive description of a large number of clonotypes of unknown specificity at a particularly point in development. We hypothesized that the complexity observed in the adults is part of a dynamic process of evolution and devolution over a lifetime (21). To examine this hypothesis, we focused on the memory component as defined by recall of the M1_58−66_-specific CD8 BV19 T cell repertoire from samples obtained from child, middle-aged, and older adult cohorts (Table 1). The repertoire consists of the canonical clonotypes whose receptor encodes Arg and Ser in the correct location of the non-germline-encoded portion of the receptor. Because we are focusing on stability as a steady-state phenomenon, time points were chosen for the recall analysis to avoid including samples after a suspected or proven influenza exposure. Immunization with trivalent flu vaccine does not appear to have an effect on the M1_58−66_ repertoire, which is not unexpected as it is not part of the vaccine.

We have previously analyzed single time points from two adult cohorts representing middle-aged and older individuals (18). These both show similar two component rank-frequency data that differ in the proportion of singleton clonotypes, lower for the older cohort, and position of critical point between components, left-shifted in older adults.

Here we present the analysis of the clonotype stability of some of the same individuals as well as others in the same age cohort and we have also provided data from a child cohort. The individual sampling data for the three age cohorts are provided in Table 1. The measures and characteristics of the recall repertories of the subjects in each of the three cohorts is provided in Supplemental Table 3 for the child cohort, Supplemental Table 4 for the middle-age, and Supplemental Table 5 for the older adult cohorts. Average values of the measures and characteristics for each cohort is provided in Supplementary Table 6.

**Figure 4:**
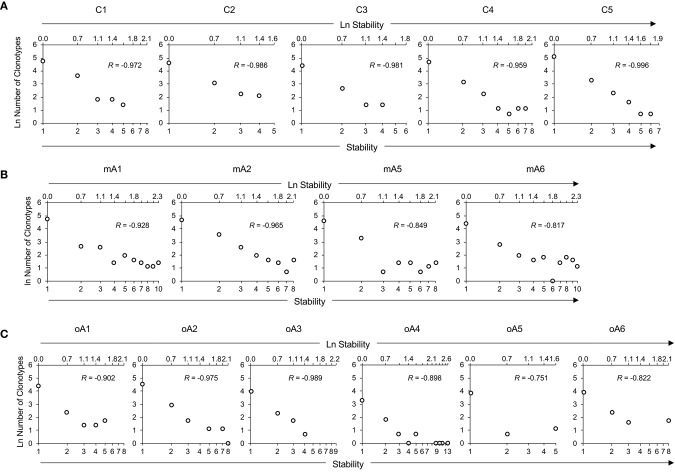
Repertoire stability data from M1_58−66_ specific recall analyses of **(A)** five child subjects, **(B)** four middle-aged adult subjects, and **(C)** of six older adult subjects. Repertoire stability data is shown as the natural log-transformed stability increment vs. number of clonotypes at that stability. The lower x-axis indicates absolute values of stability increments. The upper x-axis is demarcated in terms of the *ln* of the stability increments. Lack of a datapoint at a stability increment represents a missing value. Subject identifiers are shown above each set of panels. The *R* values represent coefficients of correlation for each dataset.

To help visualize the expected and actual outcomes of the stability analysis, the data are plotted as the natural *log* of stability, and the lower x-axis is annotated in terms of the stability increment, counting the number of sampling times in each analysis. The actual *ln* values are shown on the upper axis. A tick mark without an associated data marker represents a missing value. The BV19 RS L11 recall repertoire shows a decreased frequency of clonotypes as the stability increment increases. The regression analysis shows an excellent linear fit (*R* = −0.98 ± 0.01 and *R*^2^ = 0.96 ± 0.03), and therefore the relationship between stability and clonotype frequency can be described as power law-like (Figure 4A and Panel 1 in Table 3). Importantly, none of the five child subject repertoires had M1_58−66_-specific clonotypes that were stable; i.e., observed at all times sampled. The missing values represent the highest stability increments and the number of missing values varies from subject to subject. These data indicate that the clonotypes involved in the response are starting to show signs of increasing stability but have not yet generated a completely stable clonotypic subset of the repertoire. Even though complete stability is not attained, the stability data is described as a power law-like distribution, as was observed for the BV19 *ex vivo* adult data.

**Table 3:** Coefficients of correlation (*R*) and determination (*R*^2^) between stability and number of clonotypes for each individual within the study cohorts in reference to Figure 4.

**Children**	**Stability vs. number of clonotypes**	
	***R***	***R*^**2**^**
	**Figure 4A**	
C1	−0.972	0.945
C2	−0.986	0.972
C3	−0.981	0.962
C4	−0.959	0.920
C5	−0.996	0.992
Average^§^	−0.98 ± 0.01	0.96 ± 0.03
**Middle-aged adults**	**Figure 4B**	
mA1	−0.928	0.861
mA2	−0.965	0.931
mA5	−0.849	0.721
mA6	−0.817	0.667
Average	−0.89 ± 0.07	0.80 ± 0.12
**Older adults**	**Figure 4C**	
oA1	−0.902	0.814
oA2	−0.975	0.953
oA3	−0.989	0.979
oA4	−0.898	0.806
oA5	−0.751	0.563
oA6	−0.822	0.676
Average	−0.89 ± 0.09	0.80 ± 0.16

§ *Indicates mean ± standard deviation*.

Stability of the M1_58−66_-specific clonotypes in middle aged-subjects (Figure 4B) was similar to the BV19 *ex vivo* repertoire data, of which these clonotypes are a subset. There is power law like distribution with an increase in frequency at higher stability increments. The regression analysis shows an excellent fit of the data and a high overall correlation (*R* = −0.89 ± 0.07, Panel 2 in Table 3).

The stability data of older subjects is shown in Figure 4C and Panel 3 in Table 3. The data for oA2 is most similar to the middle-aged data. However, there are two-time point increments, 4 and 6, for which there are no values, indicating a loss of stability. Subject oA1 and oA3 can be considered to have a child-like pattern, in that there are no clonotypes present at the highest three or four stability increments. Subject oA4, oA5, and oA6 show an intermediate pattern in which there is a reversion to a child-like pattern, with the maintenance of some of the high stability pool of clonotypes. Thus, the older adult data indicates an interesting interrupted pattern in the clonotype stability pattern as would be expected from senescence of independent pools.

### Comparison of Recall Clonotype Rank Frequency and Stability as a Function of Age

**Figure 5:**
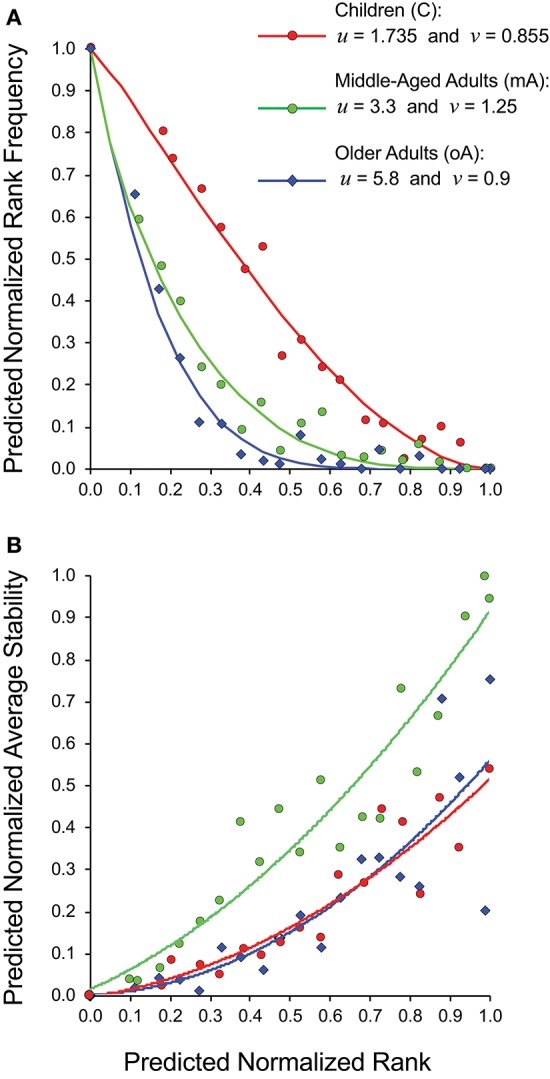
Relationship between estimated clonotype rank, rank frequency and stability of recall BV19 repertoires from child (filled circles), middle-aged adult (open circles) and older adult (diamonds) cohorts. **(A)** Predicted normalized rank vs. predicted normalized rank frequency. **(B)** Predicted normalized rank vs. predicted normalized average stability. Anchored regression lines for children (orange) and middle-aged adults (green) and older adults (blue) are shown.

Rather than trying to compare 15 panels each of rank frequency and rank stability data analyzed as five- (child), four- (middle-aged), and six- (older adult) clusters, we generated a cohort specific summary for rank vs. rank frequency (Figure 5A) and for rank vs. stability (Figure 5B). The recall repertoire data was generated by binning the normalized rank values of all subjects in a cohort in increments of 0.05 and averaging the individual rank values as well as the corresponding frequency or stability values associated with each bin. The data were fitted as anchored regressions, defined by parameters *u* and *v* using the formula, *y* = 1 – (1 – (1 – *x*)^*u*^)^*v*^. The parameter *u* controls the concave aspect of the curve, whereas *v* reflects how evenly points are distributed on the curve. With both *u* and *v* at unity, the data would constitute a straight line between (1,0) and (0,1).

For the child cohort, the parameter pair of *u* = 1.735 and *v* = 0.855 is indicative of a slight concave deviation from a 45° linear slope (Figure 5A, red circles). For the adult cohort, the pair of *u* = 3.3 and *v* = 1.25 is characteristic of a steep curvature; and for older cohort the pair of *u* = 5.8 and *v* = 0.9 is characteristic for a steady slope for the low-frequency component and a very steep curvature for a high-ranking component (Figure 5A). The values for *v* for the child cohort resemble values for the older adult cohort indicating similar properties of the low-frequency component in the repertoire distribution. The *v* value is indicative of the regularity of the rank values along the curvature; with the child cohort showing a relatively even spread, whereas the older adult values are densest in the high-frequency portion of the curve. The two adult cohort datasets show very similar patterns of distributional complexity to those we described in a previous study (18) using samples only collected at one time.

The normalized average stability values for the three cohorts (Figure 5B) shows that for the middle-aged adult cohort, the high ranking clonotypes are consistently and strongly associated with maximum stability (*R*^2^ = 0.91). The child cohort is characterized by lower stability with none of the highest ranking clonotypes were observed at all times examined (*R*^2^ = 0.85). The older adult cohort data (blue diamonds) shows a similar trendline to that of the child. However, the spread of stability values is quite wide at the high ranks (*R*^2^ = 0.71). This heterogeneity is due to individual differences within the cohort with some subjects having stable clonotypes at higher ranks while others do not (Figure 4). This is linked to the higher density of values at higher ranks in this cohort noted above. In spite of this higher density of high ranking clonotypes overall stability is lost indicating that in this cohort the relation between stability and rank is broken.

The recall data provide a focused examination of antigen-specific repertoire characteristics, but also reflect the nature of the functional definition of specificity. The data are summation of the complex distribution in the PBMC as well as the *in vitro* survival and growth potential of these cells. The latter may vary based on how many previous divisions the cells had already undergone, or requirements for costimulation that are not being met in the culture conditions. While the specific nature of the recall measurements may come at the price of additional heterogeneity, the stability and complexity measures show a definite change of a peptide-specific repertoire with age.

## Discussion

The analysis of CD8 T cell repertoire evolution presented here defines a new measurable characteristic of the circulating repertoire, clonotype stability, and shows that stable clonotypes make up a sizable fraction of the mature circulating CD8 BV19 repertoire. The symmetrical relation between the stability and rank frequency provides an explanation for the previously noted division of clonotype rank frequency into at least two distributional components when examined by rank frequency analysis. We propose that the first distributional component representing a power law-like distribution in the circulation, represents a sample of the repertoire that is sequestered in depots (bone marrow, spleen, LN, and tissues). The role of lymphocytes in the circulation has always been postulated to involve purposeful movement from memory depots to lymphoid organs or affected tissues/organs (3, 9, 10). Under normal conditions, only a small portion of circulating CD8 lymphocytes have markers indicating very recent activation (34), most express the inhibitory receptor CD31 (35), hence it is highly likely that they are being released from depots as part of a sentinel process and not in response to an exposure. We assume that the sequestered mature memory repertoire also shows a power law-like distribution. The first component would represent a proportion of the tissue/depot resident repertoire that has entered the circulation. The probability of observing the same clonotype at multiple times would be function of the frequency of that clonotype in the repertoire (its rank) and of the circulatory dwell time.

The second frequency component is over-selected in the analysis process because the continuous presence of these clonotypes in the circulation results in their being sampled at an entirely different frequency than that of the clonotypes in the more dynamic component. Clonotypes in the more stable component have been the focus of previous longitudinal HTS analyses (36, 37). With the presence of two components, examining pooled repertoires would quickly establish the stable portion but would also begin to describe the hidden portion in depots, as a cumulative sum of the dynamic, power law-like component. Ignoring or filtering out this dynamic component would provide an incomplete description of the entire repertoire.

Our data does not rule out the possibility of a second more highly expanding component that could be generated in the initial immune response. It is possible that T cell subsets each have their own reward function as described in Figure 1. There is some evidence for this possibility in the child cohort data which shows a hint of an unstable second component (Figure 5).

Our focus on canonical BV19 RS-encoding CDR3 length 11 clonotypes, does not rule out the presence of other clonotypes in children, which are supplanted by the canonical clonotypes. T cells responding to M1_58−66_ have been identified in cord-blood and blood from HLA-A2 infants, but these are no longer observed in adults (38, 39).

A circulating pool of stable clonotypes could result from the expansion of important clonotypes beyond the carrying capacity of the memory depots or tissues. We propose it represents maturation stage that maintains a quick response to recurring pathogens. This maintenance would be solidified over time as part of a robust system. It should be pointed out that a circulating depot makes sense for mature effector cells as compared to helper/regulatory T cells. Thus, it will be interesting to determine if cytotoxic CD4 cells which are often observed in mature individuals (40) also have a circulating component. It will also be interesting to define further characteristics of the stable repertoire in terms of the type of pathogen involved, whether chronic or recurring, the tissue dispersion of the pathogen, the degree of cross-reactivity of the T cells, and their surface phenotypes.

Reflection on the nature of a mature robust memory repertoire as well as our dynamic data indicate the importance of repertoire stability. Examining stability as defined here raises important issues about time and sampling, which will require further study. The BV19 *ex vivo* data are representative of short-term stability in that the elapsed time was about a year and a half. The recall data comprised a slightly longer term (Supplemental Table 1). The cohort comparisons describe the system over longer elapsed times, but these are not longitudinal. The longer the time span measured longitudinally the more confidence one has in defining a truly stable population. The key points in repertoire maturation are defining when an individual establishes a stable repertoire and when it starts to decay. These measurements are easy for the second distributional component, but more difficult for the first. In addition to frequency of timing, examination of multiple samples per time point would be useful to determine stability as defined by the sampling of a power-law like distribution in comparison to the effect of time. We expect that a careful examination of samples from older children and young adults will show evidence both for a steady buildup of clonotypes that will form the stable circulating pool as well as the transient clonotypes reflecting release from the repertoire depots.

The generation of a stable circulating component is a function of the temporal evolution of the immune system. Stable influenza-specific clonotypes were not observed in the child cohort, appeared to be well-established in middle-aged subjects, and were starting to degrade in older subjects. While our data are focused on one V family and one immune response, the self-similar nature of the system makes it likely that the observed phenomena will carry over to most CD8 T cells and responses.

**Figure 6:**
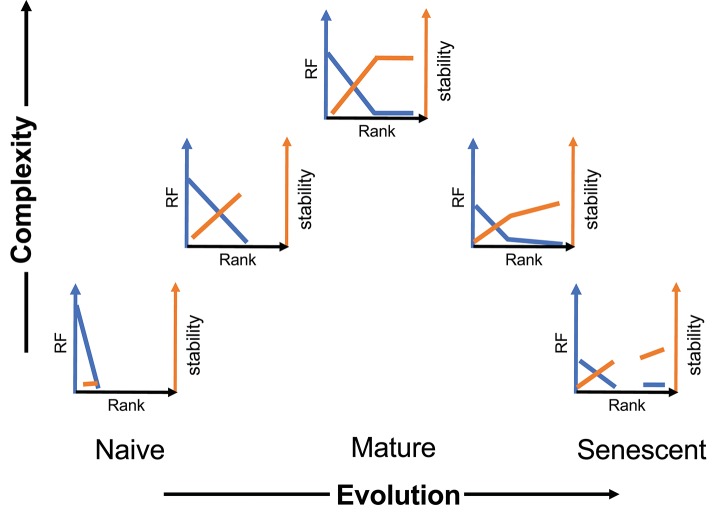
Schematic of the evolution and devolution of repertoire complexity. The y-axis represents complexity entailing both clonotype distribution (blue) and stability (orange). The extent of the complexity is representative and reflects maximum distributional and stability measures at maturity.

We present a general schematic of this temporal evolution process for CD8 cells (Figure 6) incorporating frequency and stability as a function of rank and moving left to right on a time axis and bottom to top on a complexity axis. A sample of the initial naïve repertoire (lower left panel) would be relatively uniform (mostly rank of one). It would represent a low level of complexity (although high abundance). We have previously examined the frequency of BV19, RS-encoding clonotypes in CD8 single-positive thymocytes as a proxy for the naïve repertoire, and have shown this to be the case, with a minor skewing due to the function of a rearrangement mechanism involving long P nucleotide addition from the J2-7 region (29). Upon first contacts with influenza the repertoire would show the expansion (increased rank) of selected clonotypes and an increase in stability relative to the frequency of the clonotype in the actual memory depots (second panel). With increasing number of exposures, the repertoire distribution becomes complex but does not develop the stable circulating component until maturity, which would represent the highest level of complexity (middle panel). With time and more exposures, the repertoires become more heterogeneous in their characteristics and both components of the repertoire can devolve independently.

The heterogeneity of an aging immune system is only hinted at in Figure 6. We have previously shown that even in middle-age individuals, repertoire changes in a 5- to 10-year time scale, involve a loss of the canonical BV19 RS clonotypes and an increase in other clonotypes utilized in recall responses (41). Our recall data of the older cohort here has been restricted to canonical clonotypes to aid comparison between cohorts. However, we observe a large increase in non-canonical clonotypes in recall responses from older individuals (in preparation). Our previous modeling of the changes between middle- and older-aged adult cohorts indicates an age-related loss of clonotypes based on rank (18). Re-exposure to the virus continues throughout an individual lifetime making it likely that the rank-based loss of complexity observed in the descending part of Figure 6 is due to such exposures (41). We propose that during the devolution of the repertoire there is an exposure-based loss of clonotypes, compensated by replacement with next best clonotypes, followed by the loss of the compensatory clonotypes, resulting in a tipping point synonymous with immunosenescence. Measuring the individual rate of loss from recurring exposures should provide a warning of immunosenescence and approaching criticality.

Our results describe a dynamic process of system development and aging, with increasing distributional complexity, leading to a stable circulating component, followed by loss of both complexity and stability. Along with a better understanding of the general aspects of memory generation, maintenance and decline, this study poses some fundamental questions of how well we can potentially measure T cell memory in humans and/or how complete this knowledge could be. We still have no answers to how frequently and for how long we should measure a repertoire in order to define its stability. Could a routine blood sample be a representative sample of the circulating pool? And if not, what is the alternative. We expect that stability will be affected by pathogen exposure, hence our care in trying to eliminate that aspect from the current analysis. But what degree of departure from stability would be considered as a measure of resilience or decline? These emergent questions are immediately important in thinking about circulating cells as a source for immunomodulatory therapy and they shape a new direction in quantification of the way immune memory evolves.

## Data Availability

The raw data supporting the conclusions of this manuscript will be made available by the authors, without undue reservation, to any qualified researcher. The curated dataset used for the analyses here are provided as Supplemental Data 1.

## Ethics Statement

The healthy child subjects were enrolled under protocol CHW IRBnet: 116305 Generation and decay of memory T cells in children with Juvenile Rheumatoid Arthritis and healthy siblings following administration of trivalent inactivated influenza vaccine, from the Children Hospital of Wisconsin. The subjects analyzed here were the controls in this study. Written informed consent was obtained from participants, or their parents/legal guardians in the case of children. The adult subjects were enrolled under protocols authorized by the Institutional Review Board of BloodCenter of Wisconsin: BC 05-11, Generation and Decay of Memory T Cells in Older Populations, and BC 04-22, Robust T Cell Immunity to Influenza in Human Populations. These protocols have been transferred to the IRB of the Medical College of Wisconsin (MCW).

## Author Contributions

All authors have read and approved the manuscript. EN implemented and performed the high-level analyses and participated in writing the paper. MY was involved in both the *ex vivo* and recall analyses, and in organizing the experiments. WD was involved in the *ex vivo* analyses. ER and MU generated the recall data for the adult cohorts. DH generated the recall repertoire and MU analyzed the clonotypes in the child cohort. CW had overall responsibility for the child cohort analyses. YN was involved in analyzing the recall repertoire in the adults, and in data analysis. JG was responsible for the overall study design and writing the paper.

### Conflict of Interest Statement

The authors declare that the research was conducted in the absence of any commercial or financial relationships that could be construed as a potential conflict of interest.

## Supplementary Material

Supplemental Figure 1 Clonotype naming algorithm. The name is meant to be available for computation. It consists of the concatenation of the NDN amino acids in single letter code, in upper case, proceeded and followed by the last amino acid completely V-encoded and the first completely J encoded in lower case. A period separates the amino acids from the NDN encoding. This is followed by the V identifier which starts with B or A for beta or alpha followed by the family number, the letter “s” and the subfamily identifier. Next come the J-region identification, with again A for alpha, B for beta. Followed by the J cluster and locus position identifier, with no separator. The name provides the information as to the key diversity element that distinguishes this V-J combination from any other using the exact same V and J. To regenerate the CDR3 nucleotide sequence from the name one moves backwards through the naming algorithm. **(A)** We start with the clonotype name of the most frequent clonotype in the C1 recall dataset. The key data are colored to identify their content. **(B)** The genomic sequences of the V and J are lined up so that there are L codons between the Cys and Phe Gly. The Amino acids of the NDN (upper case) are aligned and the nucleotide sequence of each is inserted. **(C)** The nucleotide sequence is assembled by overlapping the V, NDN, and J sequences with the V and J lower case base pairs being used first until they no longer match the NDN codons. The germline contribution to the CDR3 is underlined in the sequences provided in **(B)**. This provides the best estimate of the rearrangement point. **(D)** The subset of the genetic code table needed for converting the a.a. name and encoding to nucleotide sequence in step B. The period in the name provides a visual break, but is also useful to FIND the start of the codon ID string. The NDN region can be substringed by starting at position 2 and stopping before the period. The NDN length can be determined from the same procedure. The “L” provides a visual break between the J and CDR3 length numerical identification. The letter “S” was used at one time to separate the V family and Subfamily identification. Current nomenclature uses a bar. Current nomenclature does not require a TRBV19-1 since there is no 19-2 locus. However we include the subfamily tag to maintain spacing in the name. All lengths take two characters, hence a CDR3 length of 9 amino acids is L09. Alleles are neglected. There are no V alleles reported yet in the region distal to the Cys. There are two J-region alleles, one is silent, but the one in J2-7 changes the F in the FG to V. The nomenclature could be modified to include allele identification but it would be wise to avoid the current nomenclature use of an asterisk as it is usually interpreted as a wild card and requires additional handling if the name is being used in a computation.

Supplemental Table 1 Time-map of peripheral blood collection for each subject relative to first sampling.

Supplemental Table 2 Summary of high-throughput sequencing data for the adult cohort.

Supplemental Table 3 Measures and characteristics of the M_158−66_-specific recall repertoires for the child cohort.

Supplemental Table 4 Measures and characteristics of the M_158−66_-specific recall repertoires for the middle-aged adult cohort.

Supplemental Table 5 Measures and characteristics of the M_158−66_-specific recall repertoires for the older adult cohort.

Supplemental Table 6 Summary of M_158−66_-specific recall BV19 repertoire measures and characteristics for the child, middle-aged, and older adult cohorts.

Supplemental Data 1 The clonotype names, counts, and stability values for all the analyses presented in this manuscript are provided.
