# Supplementary figures and images for: Age-Based Dynamics of a Stable Circulating Cd8 T Cell Repertoire Component
Source: Front Immunol. 2019 Aug 6;10:1717. doi: 10.3389/fimmu.2019.01717 (PMC6691812; doi:10.3389/fimmu.2019.01717)

Supplemental Figure 1

Clonotype name convention

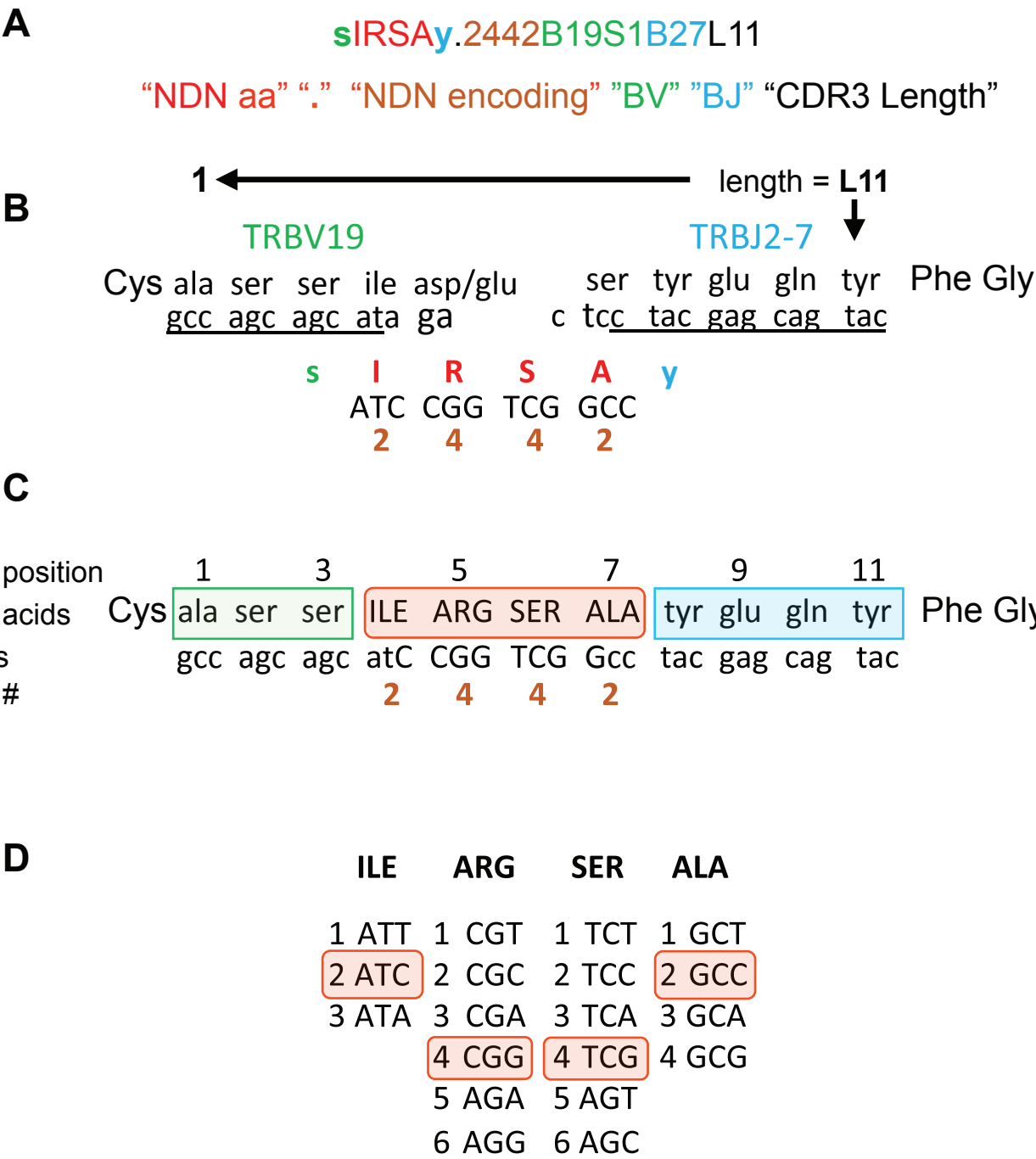

Supplement: Supplemental Figure 1 — Clonotype naming algorithm. The name is meant to be available for computation. It consists of the concatenation of the NDN amino acids in single letter code, in upper case, proceeded and followed by the last amino acid completely V-encoded and the first completely J encoded in lower case. A period separates the amino acids from the NDN encoding. This is followed by the V identifier which starts with B or A for beta or alpha followed by the family number, the letter “s” and the subfamily identifier. Next come the J-region identification, with again A for alpha, B for beta. Followed by the J cluster and locus position identifier, with no separator. The name provides the information as to the key diversity element that distinguishes this V-J combination from any other using the exact same V and J. To regenerate the CDR3 nucleotide sequence from the name one moves backwards through the naming algorithm. (A) We start with the clonotype name of the most frequent clonotype in the C1 recall dataset. The key data are colored to identify their content. (B) The genomic sequences of the V and J are lined up so that there are L codons between the Cys and Phe Gly. The Amino acids of the NDN (upper case) are aligned and the nucleotide sequence of each is inserted. (C) The nucleotide sequence is assembled by overlapping the V, NDN, and J sequences with the V and J lower case base pairs being used first until they no longer match the NDN codons. The germline contribution to the CDR3 is underlined in the sequences provided in (B). This provides the best estimate of the rearrangement point. (D) The subset of the genetic code table needed for converting the a.a. name and encoding to nucleotide sequence in step B. The period in the name provides a visual break, but is also useful to FIND the start of the codon ID string. The NDN region can be substringed by starting at position 2 and stopping before the period. The NDN length can be determined from the same procedure. The “L” provides a [file Image_1.pdf]
