# Supplementary material for: Age-Based Dynamics of a Stable Circulating Cd8 T Cell Repertoire Component
Source: Front Immunol. 2019 Aug 6;10:1717. doi: 10.3389/fimmu.2019.01717 (PMC6691812; doi:10.3389/fimmu.2019.01717)
Supplement: Supplemental Table 1 — Time-map of peripheral blood collection for each subject relative to first sampling. [file Table_1.pdf]

Supplemental Table 1. Time-map of peripheral blood collection for each subject relative to first sampling

	RECALL															*ex vivo* HTS				
	Children					Middle-aged Adults				Older Adults						Adults				
**ID**	**C1**	**C2**	**C3**	**C4**	**C5**	**mA1**	**mA2**	**mA5**	**mA6**	**oA1**	**oA2**	**oA3**	**oA4**	**oA5**	**oA6**	**oA1**	**mA1**	**mA2**	**mA3**	**mA4**
**First Sample Mon-YY**	Oct-07	Jun-07	Nov-07	Oct-07	Dec-07	Dec-04	Jul-05	Apr-05	Apr-05	Dec-05	Nov-05	Nov-05	Apr-06	Jan-07	Dec-05	Aug-06	Mar-06	Jul-05	Oct-08	Jun-06
**Elapsed time in months**																				
0	x	x	x	x	x	x	x	x	x	x	x	x	x	x	x	x	x	x	x	x
1	x		x	x																
2						x		x	x	x						x	x		x	
3							x				x		x					x		
4								x		x										
5	x			x	x		x											x		
6			x			x		x		x	x		x			x	x		x	x
7															x					
8								x	x	x		x	x	x		x	x		x	x
9						x					x						x			
10										x		x	x						x	
11					x	x		x	x											x
12	x	x	x	x						x	x		x		x					
13							x	x							x	x		x		
14						x	x			x		x		x				x		x
15	x			x	x								x		x					
16			x			x					x	x					x			
17		x						x									x	x		x
18					x	x			x			x		x						
19			x				x				x		x			x				
20	x			x		x			x											
21												x	x							
22		x			x		x		x					x						
23					x	x							x		x					
24							x													
25	x			x									x							
26	x			x					x			x								
27																				
28												x			x					
29																				
30																				
31																				
32		x													x					
33									x											
34																				
35																				
36													x							
37																				
38																				
39									x											
40											x									
41																				
42																				
43																				
44																				
45																				
46													x							

ID - subject unique identification marker
x - blood sample collection
