# Supplementary material for: Age-Based Dynamics of a Stable Circulating Cd8 T Cell Repertoire Component
Source: Front Immunol. 2019 Aug 6;10:1717. doi: 10.3389/fimmu.2019.01717 (PMC6691812; doi:10.3389/fimmu.2019.01717)
Supplement: Supplemental Table 2 — Summary of high-throughput sequencing data for the adult cohort. [file Table_2.pdf]

Supplemental Table 2. Summary of high-throughput sequencing data for the adult cohort.

	Subject ID				
**Pooled Repertoires**	**oA1**	**mA1**	**mA2**	**mA3**	**mA4**
Number of unique clonotypes, N	12269	4251	10921	13348	22708
Number of observations, M	203183	149882	234800	188963	240294
Number of singleton clonotypes, N_S	1835	1026	2968	2471	5572
Number of observations of the most frequent clonotype (maximum rank), $Rmax$	5782	58904	9871	31258	23326
Number of stable clonotypes, N_{st}	191	88	56	81	97
Number of observations of stable clonotypes, M_{st}	83234	53608	71582	65723	95696
Proportion of singletons observations, $Ps = \frac{Ns}{M}$	0.009	0.007	0.013	0.013	0.023
Proportion of observations of the most frequent clonotype, $Pmax = \frac{Rmax}{M}$	0.028	0.393	0.042	0.165	0.097
Fraction of singletons, $\frac{Ns}{N}$	0.150	0.241	0.272	0.187	0.245
Average number of observations per clonotype, $V = \frac{M}{N}$	16.56	35.26	21.50	14.16	10.58
Proportion of stable clonotypes, $P_{Mst} = \frac{Mst}{M}$	0.410	0.751	0. 305	0.348	0.398
Fraction of stable clonotypes, $\frac{Nst}{N}$	0.016	0.021	0.005	0.006	0.004
Clonotype diversity, $Dc = \frac{Rmax\,N}{M} - 1$	348.1	1669.7	458.1	2207.0	2203.3
**Average of Individual Repertoires in Pool** (mean ± standard deviation) §					
Number of all unique clonotypes, N	2566.00 ± 390.55	858.14 ± 226.65	2024.50 ± 1841.32	2958.00 ± 1344.05	4185.17 ± 2926.75
Number of observations, M	33863 ± 11876	21412 ± 3632	39133 ± 18999	37793 ± 6667	40049 ± 4786
Number of all singletons, N_S	393.83 ± 71.03	190.71 ± 48.17	551.83 ± 377.23	581.20 ± 203.22	1021.67 ± 774.45
Number of observations of the most frequent clonotype (maximum rank), $Rmax$	1197.17 ± 513.52	8414.86 ± 2663.33	1895.17 ± 1967.83	6251.60 ± 1951.67	3887.67 ± 2088.81
Proportion of singletons observations, $Ps = \frac{Ns}{M}$	0.013 ± 0.006	0.009 ± 0.003	0.015 ± 0.006	0.016 ± 0.008	0.026 ± 0.020
Proportion of observations of the most frequent clonotype, $Pmax = \frac{Rmax}{M}$	0.04 ± 0.01	0.39 ± 0.10	0.04 ± 0.029	0.16 ± 0.03	0.10 ± 0.04
Fraction of singletons, $\frac{Ns}{N}$	0.16 ± 0.03	0.23 ± 0.04	0.30 ± 0.08	0.21 ± 0.04	0.24 ± 0.03
Average number of observations per clonotype, $V = \frac{M}{N}$	13.08 ± 3.21	26.31 ± 7.04	24.81 ± 14.98	15.29 ± 7.47	15.03 ± 9.66
Clonotype diversity, $Dc = \frac{Rmax\,N}{M} - 1$	89.61 ± 30.06	320. 64 ± 63.01	70.99 ± 50.07	473.16 ± 254.84	314.24 ± 154.75

§ - Number of samples collected per subject is given in Table 1
