# Supplementary material for: Age-Based Dynamics of a Stable Circulating Cd8 T Cell Repertoire Component
Source: Front Immunol. 2019 Aug 6;10:1717. doi: 10.3389/fimmu.2019.01717 (PMC6691812; doi:10.3389/fimmu.2019.01717)
Supplement: Supplemental Table 3 — Measures and characteristics of the M158−66-specific recall repertoires for the child cohort. [file Table_3.pdf]

Supplemental Table 3. Measures and characteristics of the $M1_{58\text{-}66}$-specific recall repertoires for the child cohort.

	Subject ID				
**Pooled Repertoires**	**C1**	**C2**	**C3**	**C4**	**C5**
Number of all unique clonotypes, *N*	166	133	99	147	204
Number of observations of all unique clonotypes, *M*	826	514	574	723	935
Number of all singletons, N_S	67	57	32	56	84
Number of observations of the most frequent clonotype (maximum rank), *Rmax*	44	31	36	59	63
Proportion of singletons observations, $Ps = \frac{Ns}{M}$	0.081	0.111	0.056	0.077	0.090
Proportion of observations of the most frequent clonotype, $Pmax = \frac{Rmax}{M}$	0.053	0.060	0.063	0.082	0.067
Fraction of singletons, $\frac{Ns}{N}$	0.404	0.429	0.323	0.381	0.412
Average number of observations per clonotype, $V = \frac{M}{N}$	4.98	3.86	5.80	4.92	4.58
Clonotype diversity, $Dc = \frac{Rmax\,N}{M} - 1$	7.84	7.02	5.21	11.00	12.75
**Average of Individual Repertoires in Pool** (mean ± standard deviation) §					
Number of all unique clonotypes, *N*	31.00 ± 5.78	39.20 ± 6.02	22.83 ± 10.17	29.75 ± 8.41	40.43 ± 9.48
Number of observations, *M*	103.25 ± 13.65	102.80 ± 14.06	95.67 ± 14.35	90.38 ± 22.05	133.57 ± 16.30
Number of all singletons, N_S	14.63 ± 5.78	19.80 ± 2.95	7.67 ± 4.41	13.63 ± 5.60	17.86 ± 5.73
Number of observations of the most frequent clonotype (maximum rank), *Rmax*	20.63 ± 6.02	12.80 ± 4.21	20.50 ± 7.40	15.13 ± 5.17	26.43 ± 14.42
Proportion of singletons observations, $Ps = \frac{Ns}{M}$	0.15 ± 0.07	0.19 ± 0.01	0.08 ± 0.04	0.15 ± 0.05	0.14 ± 0.05
Proportion of observations of the most frequent clonotype, $Pmax = \frac{Rmax}{M}$	0.21 ± 0.08	0.13 ± 0.04	0.22 ± 0.10	0.17 ± 0.06	0.19 ± 0.10
Fraction of singletons, $\frac{Ns}{N}$	0.46 ± 0.13	0.51 ± 0.04	0.31 ± 0.09	0.44 ± 0.11	0.44 ± 0.08
Average number of observations per clonotype, $V = \frac{M}{N}$	3.43 ± 0.72	2.64 ± 0.27	4.84 ± 1.95	3.10 ± 0.42	3.45 ± 0.87
Clonotype diversity $Dc = \frac{Rmax\,N}{M} - 1$,	5.08 ± 1.52	3.76 ± 1.23	3.31 ± 0.71	3.95 ± 1.82	6.17 ± 2.56

§ Number of samples collected per subject is given in Table 1
