# Supplementary material for: Age-Based Dynamics of a Stable Circulating Cd8 T Cell Repertoire Component
Source: Front Immunol. 2019 Aug 6;10:1717. doi: 10.3389/fimmu.2019.01717 (PMC6691812; doi:10.3389/fimmu.2019.01717)
Supplement: Supplemental Table 4 — Measures and characteristics of the M158−66-specific recall repertoires for the middle-aged adult cohort. [file Table_4.pdf]

Supplemental Table 4. Measures and characteristics of the $M1_{58\text{-}66}$-specific recall repertoires for the middle-aged adult cohort.

	Subject ID			
**Pooled Repertoires**	**mA1**	**mA2**	**mA5**	**mA6**
Number of all unique clonotypes, N	173	176	146	136
Number of observations, M	2449	3015	2128	3166
Number of all singletons, N_S	87	45	65	69
Number of observations of the most frequent clonotype (maximum rank), $Rmax$	935	168	1171	292
Proportion of singletons observations, $Ps = \frac{Ns}{M}$	0.036	0.015	0.031	0.022
Proportion of observations of the most frequent clonotype, $Pmax = \frac{Rmax}{M}$	0.382	0.056	0.550	0.092
Fraction of singletons, $\frac{Ns}{N}$	0.503	0.256	0.445	0.507
Average number of observations per clonotype, $V = \frac{M}{N}$	14.16	17.13	14.58	23.28
Clonotype diversity, $Dc = \frac{Rmax\,N}{M} - 1$	65.05	8.81	79.34	11.54
**Average of Individual Repertoires in Pool** (mean ± standard deviation) §				
Number of all unique clonotypes, N	38.30 ± 11.71	43.25 ± 12.34	32.50 ± 20.28	34.30 ± 6.67
Number of observations, M	224.90 ± 122.38	376.88 ± 36.81	266.00 ± 91.01	316.60 ± 51.68
Number of all singletons, N_S	16.40 ± 8.25	12.13 ± 5.69	14.63 ± 12.28	12.20 ± 5.71
Number of observations of the most frequent clonotype (maximum rank), $Rmax$	95.70 ± 57.59	56.50 ± 16.49	146.38 ± 78.43	64.30 ± 27.14
Proportion of singletons observations, $Ps = \frac{Ns}{M}$	0.07 ± 0.03	0.03 ± 0.02	0.06 ± 0.05	0.04 ± 0.02
Proportion of observations of the most frequent clonotype, $Pmax = \frac{Rmax}{M}$	0.39 ± 0.15	0.15 ± 0.04	0.53 ± 0.17	0.20 ± 0.07
Fraction of singletons, $\frac{Ns}{N}$	0.41 ± 0.11	0.27 ± 0.06	0.38 ± 0.15	0.34 ± 0.13
Average number of observations per clonotype, $V = \frac{M}{N}$	6.50 ± 3.11	9.34 ± 2.74	12.79 ± 11.06	9.57 ± 2.46
Clonotype diversity, $Dc = \frac{Rmax\,N}{M} - 1$	13.66 ± 6.97	5.07 ± 0.62	13.69 ± 6.36	5.77 ± 2.36

§ - Number of samples collected per subject is given in Table 1
