# Supplementary material for: Age-Based Dynamics of a Stable Circulating Cd8 T Cell Repertoire Component
Source: Front Immunol. 2019 Aug 6;10:1717. doi: 10.3389/fimmu.2019.01717 (PMC6691812; doi:10.3389/fimmu.2019.01717)
Supplement: Supplemental Table 5 — Measures and characteristics of the M158−66-specific recall repertoires for the older adult cohort. [file Table_5.pdf]

Supplemental Table 5. Measures and characteristics of the $M1_{58-66}$-specific recall repertoires for the older adult cohort.

	**Subject ID**					
**Pooled Repertoires**	**oA1**	**oA2**	**oA3**	**oA4**	**oA5**	**oA6**
Number of all unique clonotypes, N	106	130	72	41	52	74
Number of observations, M	1983	2437	2637	2103	1350	2041
Number of all singletons, N_S	41	60	38	13	28	24
Number of observations of the most frequent clonotype (maximum rank), $Rmax$	172	776	537	934	524	621
Proportion of singletons observations, $Ps = \frac{Ns}{M}$	0.021	0.025	0.014	0.006	0.021	0.012
Proportion of observations of the most frequent clonotype, $Pmax = \frac{Rmax}{M}$	0.087	0.318	0.204	0.444	0.388	0.304
Fraction of singletons, $\frac{Ns}{N}$	0.387	0.462	0.528	0.317	0.538	0.324
Average number of observations per clonotype, $V = \frac{M}{N}$	18.71	18.78	36.63	51.29	25.96	27.58
Clonotype diversity, $Dc = \frac{Rmax\,N}{M} - 1$	8.19	40.40	13.66	17.21	19.18	21.52
**Average of Individual Repertoires in Pool** (mean ± standard deviation) §						
Number of all unique clonotypes, N	20.13 ± 3.36	24.75 ± 9.50	11.11 ± 5.90	7.77 ± 3.17	13.20 ± 4.97	17.13 ± 2.85
Number of observations, M	247.88 ± 72.44	304.63 ± 96.09	293.00 ± 116.78	161.77 ± 38.38	270.00 ± 147.34	255.13 ± 25.45
Number of all singletons, N_S	6.88 ± 3.27	10.88 ± 7.70	5.22 ± 4.27	1.85 ± 1.28	5.60 ± 3.44	5.25 ± 1.16
Number of observations of the most frequent clonotype (maximum rank), $Rmax$	76.88 ± 30.15	164.13 ± 116.80	167.56 ± 125.39	77.62 ± 29.71	139.00 ± 112.69	78.50 ± 20.92
Proportion of singletons observations, $Ps = \frac{Ns}{M}$	0.03 ± 0.03	0.03 ± 0.03	0.02 ± 0.02	0.01 ± 0.01	0.03 ± 0.02	0.02 ± 0.01
Proportion of observations of the most frequent clonotype, $Pmax = \frac{Rmax}{M}$	0.31 ± 0.08	0.50 ± 0.25	0.52 ± 0.20	0.49 ± 0.17	0.50 ± 0.21	0.31 ± 0.09
Fraction of singletons, $\frac{Ns}{N}$	0.34 ± 0.14	0.39 ± 0.22	0.44 ± 0.17	0.23 ± 0.16	0.39 ± 0.15	0.31 ± 0.09
Average number of observations per clonotype, $V = \frac{M}{N}$	12.81 ± 5.19	13.39 ± 4.43	32.62 ± 19.94	24.94 ± 14.90	28.62 ± 31.61	15.14 ± 2.26
Clonotype diversity $Dc = \frac{Rmax\,N}{M} - 1$,	5.13 ± 1.50	10.57 ± 7.00	4.38 ± 2.61	2.37 ± 0.92	4.79 ± 1.81	4.14 ± 1.04

§ - Number of samples collected per subject is given in Table 1
