# Supplementary material for: Age-Based Dynamics of a Stable Circulating Cd8 T Cell Repertoire Component
Source: Front Immunol. 2019 Aug 6;10:1717. doi: 10.3389/fimmu.2019.01717 (PMC6691812; doi:10.3389/fimmu.2019.01717)
Supplement: Supplemental Table 6 — Summary of M158−66-specific recall BV19 repertoire measures and characteristics for the child, middle-aged, and older adult cohorts. [file Table_6.pdf]

Supplemental Table 6. Summary of $M1_{58\text{-}66}$-specific recall BV19 repertoire measures and characteristics for the child, middle-aged and older adult cohorts.

**Cohort Measures and Characteristics** §	**Children**	**Middle-Aged Adults**	**Older Adults**
Number of all unique clonotypes, N	150 ± 39	158 ± 20	79 ± 33
Number of observations of all unique clonotypes, M	714 ± 174	2690 ± 485	2092 ± 443
Number of all singletons, N_S	59 ± 19	67 ± 17	34 ± 16
Maximum number of observations per a unique clonotype, $Rmax$	47 ± 14	642 ± 487	594 ± 259
Proportion of singletons observations, $Ps = \frac{Ns}{M}$	0.083 ± 0.020	0.026 ± 0.009*	0.016 ± 0.007**
Proportion of observations of the most frequent clonotype, $Pmax = \frac{Rmax}{M}$	0.065 ± 0.011	0.270 ± 0.237	0.291 ± 0.129**
Fraction of singletons, $\frac{Ns}{N}$	0.390 ± 0.041	0.428 ± 0.118	0.426 ± 0.098
Average number of observations per clonotype, $V = \frac{M}{N}$	4.828 ±0.700	17.285 ± 4.207*	29.819 ± 12.437**
Clonotype diversity, $Dc = \frac{Rmax\, N}{M} - 1$	8.763 ± 3.056	41.185 ± 36.297	20.027 ± 11.011
Correlation between stability and number of clonotypes	-0.979 ± 0.014	-0.890 ± 0.069	-0.890 ± 0.091
Correlation between normalized rank and normalized rank frequency	-0.912 ± 0.028	-0.747 ± 0.090*	-0.673 ± 0.041**
Correlation between normalized rank and normalized average stability	0.647± 0.132	0.810 ± 0.078	0.657 ± 0.135

§ - indicates mean ± standard deviation
* - indicates significant differences between the child and adult cohorts with p value < 0.05 for t-test.
** - indicates significant differences between the child and older adult cohorts with p value < 0.05 for t-test.
